# Absence of orthopaedia homeobox protein (OTP) expression is associated with disease spread and adverse outcome in pulmonary carcinoid tumour patients

**DOI:** 10.1007/s00428-024-03847-z

**Published:** 2024-06-19

**Authors:** Jenni Niinimäki, Sanna Mononen, Tuomas Kaprio, Johanna Arola, Tiina Vesterinen

**Affiliations:** 1 Department of Pathology, University of Helsinki and Helsinki University Hospital, Haartmaninkatu 3, 00014 Helsinki, Finland; 2 Department of Surgery, Helsinki University Hospital, Helsinki, Finland; 3 Translational Cancer Medicine Research Program, Faculty of Medicine, University of Helsinki, Helsinki, Finland; 4 HUS Diagnostic Center, HUSLAB, Department of Pathology, University of Helsinki and Helsinki University Hospital, Helsinki, Finland

**Keywords:** Pulmonary carcinoid, Neuroendocrine tumour, Orthopaedia homeobox protein (OTP), Prognostics, Immunohistochemistry

## Abstract

Pulmonary carcinoid (PC) tumours typically have a good prognosis, although metastases occur, and the disease may progress after a long period of time. Expression of orthopaedia homeobox protein (OTP) has been recognized as a possible independent prognostic marker in PCs. Immunohistochemical (IHC) OTP expression has been associated with better prognosis, but the staining has yet to be implemented in routine clinical diagnostics. In response to this, two new monoclonal OTP antibodies were recently developed.

This retrospective study included 164 PC patients operated on at Helsinki University Hospital between 1990 and 2020. Tissue microarray slides, prepared from formalin-fixed and paraffin-embedded primary tumour samples, were stained with OTP IHC using one polyclonal and two novel monoclonal antibodies.

Absence of OTP expression was associated with a shorter disease-specific survival (DSS) and disease progression (*p* < 0.001). Patients without OTP expression had a 5-year DSS of 73–79%, whereas 5-year DSS was 91–94% with OTP expression, depending on the primary antibody. In a univariable Cox regression model, absence of OTP expression was associated with adverse outcome along with atypical histological subtype, metastatic disease, Ki-67 proliferation index > 1%, and larger tumour size. In a multivariable Cox regression model, only absence of OTP expression and lymph node involvement at the time of diagnosis were associated with risk of worse prognosis. All three antibodies showed good concordance with each other.

Our findings support the role of OTP as an independent prognostic marker in PCs and applicability of IHC staining in routine clinical use with novel monoclonal antibodies.

**Supplementary Information:**

The online version contains supplementary material available at 10.1007/s00428-024-03847-z.

## Introduction

Pulmonary carcinoids (PCs) are rare lung neoplasms of neuroendocrine origin. Lung neuroendocrine neoplasms constitute a spectrum of diseases varying greatly in clinical behaviour and prognosis. They are classified as high-grade (small cell lung carcinoma and large cell neuroendocrine carcinoma), intermediate-grade (atypical carcinoid), and low-grade (typical carcinoid) tumours. The incidence of lung carcinoid tumours is reported to be as low as 0.2–2 per 100000 per year, but the incidence is rising [1, 2]. In terms of lung cancer in general, PCs are rare, constituting 1–2% of all malignant lung tumours [2, 3].

Typical carcinoids (TCs) are well-differentiated low-grade neuroendocrine tumours. Reflecting the low-grade malignant potential of TCs, long-term survival from these tumours is favorable, 80–100%, especially when resected [4–6]. Atypical carcinoids (ACs) are considered intermediate-grade tumours. Compared with TCs, their prognosis is less favorable but still good, with a five-year survival of 40–90% [4–6]. This wide range in reported survival could be explained at least in part by differences in prognosis according to disease stage and applicability of surgical treatment [2, 4, 5]. Furthermore, ACs are rare even in terms of PCs [2, 3], which causes heterogeneity in survival results between different studies. Radical surgery is the main method of treatment for PCs and the only curative option. Histologically, TCs are distinguished from ACs by the absence of necroses and lower mitotic count, less than 2 mitoses per 2 mm^2^ [2, 3].

Although the prognosis is considered good, especially with TC, metastatic disease and/or disease progression are encountered with both TC and AC. Current guidelines recommend at least 10 years of follow-up [7], or an approach based on risk assessment [1]. Thus, recognizing patients at higher risk for disease progression could help identify those benefiting from long-term follow-up. In addition to histological subtype (TC vs. AC), TNM classification, tumour size, nodal status, metastases, Ki-67 proliferation index (PI), mitotic index, age, sex, and performance status have been variably reported as prognostic factors [2, 4–6].

In the current version of the World Health Organization (WHO) classification, a member of the homeobox protein family, orthopaedia homeobox protein (OTP) is listed as a possible prognostic marker along with the adhesion molecule CD44 [2]. In normal tissue, the exact function of OTP is largely unknown, although it has been considered an important factor in the developing hypothalamic neuroendocrine system in vertebrates [8]. However, in 2013, Swartz et al. published two studies in which the downregulation of *OTP* at the individual gene level and the absence of its expression at the protein level were found to be related to an unfavourable disease outcome in PC tumour patients [9, 10]. Since then, several studies have investigated the potential of immunohistochemical OTP expression to distinguish aggressive carcinoid tumours from indolent ones [11–14].

Among patients with resected PC tumour, nuclear OTP expression has been associated with better outcomes [10–14]. OTP expression has been shown to be associated with different levels of DNA methylation in PCs, suggesting a possible mechanism by which OTP expression affects the behaviour of these tumours [15]. Moreover, an association between OTP and thyroid transcription factor 1 (TTF1), a common immunohistochemical marker used in PC diagnostics, has been proposed [16]. In addition to prognostication, OTP has been suggested as a possible diagnostic marker in differentiating PCs from other pulmonary neoplasms [16–18] or from neuroendocrine tumours of other than pulmonary origin [16, 18, 19]. OTP has also been found to be expressed in diffuse idiopathic pulmonary neuroendocrine cell hyperplasia (DIPNECH), which is considered a precursor of a subset of PCs, but not in normal neuroendocrine cells of the lung, suggesting a possible role in PC development [16].

In most of the previous studies, a rabbit polyclonal OTP primary antibody (HPA039365, Atlas Antibodies, Stockholm, Sweden) was used for immunohistochemical staining. However, the discontinuation of this antibody and the lack of similarly performing options have hindered implementation of OTP staining in routine diagnostics. Recently, two commercially produced monoclonal OTP primary antibodies (Atlas Antibodies CL11222, CL11225) were developed, verified, and released [20].

The aim of our study was to evaluate the association of OTP expression with disease progression and disease-specific survival in PC tumour patients. Moreover, we aimed to compare the performance of the new monoclonal antibodies with the previously used polyclonal antibody.

## Materials and methods

### Patients

A total of 164 PC tumour patients operated on between 1990 and 2020 at Helsinki University Hospital, Finland, were included in this retrospective study. Most of these tumours were TCs (*n* = 123), and the rest were ACs (*n* = 41). In all, 30 of the tumours had metastasized, and of these, 20 showed lymph node involvement at the time of diagnosis. Formalin-fixed and paraffin-embedded (FFPE) primary tumour samples were retrieved through the Helsinki Biobank. Associated clinical information was collected from patient records (Table 1).

**Table 1:** Clinicopathological characteristics of the patients

Variable	TC (*n* = 123)		AC (*n* = 41)		All (*n* = 164)	
Sex, *n* (%)						
Male	38	(31)	20 20	(49)	58	(35)
Female	85	(69)	21	(51)	106	(65)
Age (years)						
mean	55		56		55	
median	57		59		58	
range	19–84		17–83		17–84	
Tumour size (cm), *n* (%)						
≤ 1	33	(28)	10	(25)	43	(27)
1.1–2.9	69	(57)	20	(50)	89	(56)
≥ 3	18	(15)	10	(25)	28	(17)
Not available	3		1		4	
Nodal involvement at diagnosis, *n* (%)						
Yes	10	(11)	10	(26)	20	(15)
No	83	(89)	28	(74)	111	(85)
Not examined	30		3		33	
Distant metastasis, *n* (%)						
At diagnosis	0	(0)	3	(7)	3	(2)
During follow-up	10	(8)	8	(20)	18	(11)
Surgical procedure, *n* (%)						
Lobectomy	60	(55)	22	(56)	82	(55)
Sleeve resection	15	(14)	8	(20)	23	(16)
Segmentectomy	16	(15)	2	(5)	18	(12)
Wedge resection	12	(11)	1	(3)	13	(9)
Bilobectomy	1	(1)	2	(5)	3	(2)
Enucleation	4	(3)	1	(3)	5	(3)
Pneumoectomy	1	(1)	3	(8)	4	(3)
Not available	14		2		16	
Neoadjuvant treatment, *n*						
Chemotherapy	0		0		0	
Radiotherapy	0		1		1	
Both	0		1		1	
Adjuvant treatment ^a^, *n*						
Chemo/radiotherapy only	4		1		5	
Chemo + radiotherapy	1		3		4	
SSAs only	3		0		3	
SSAs + chemo/radiotherapy (or both)	2		2		4	
SSAs + PRRT	1		1		2	
SSAs + PRRT +	1		2		3	
chemo/radiotherapy						
Ki-67 proliferation index, *n* (%)						
< 1%	37	(30)	13	(32)	50	(30)
1–2%	77	(63)	19	(46)	96	(59)
> 2%	9	(7)	9	(22)	18	(11)

^a^, Including treatment of metastatic disease. *TC*, typical carcinoid; *AC*, atypical carcinoid; *SSA*, somatostatin analog; *PRRT*, peptide receptor radionuclide therapy

Each tumour was re-evaluated from diagnostic whole slides by an experienced pulmonary pathologist following the 2021 classification of pulmonary neuroendocrine tumours by the WHO [2]. Neuroendocrine differentiation and epithelial origin were confirmed by routine immunohistochemical labelling for chromogranin A, synaptophysin, and pan-cytokeratin.

This study was approved by the HUS Regional Committee on Medical Research Ethics (HUS/1258/2020). As the Finnish Biobank Act provides a lawful basis for research use of patient samples and clinical data, a project-specific consent was not obtained from the patients who had given a biobank consent (*n* = 146). Eighteen patients had not given a biobank consent, but their samples and data were used based on the statements of the Finnish Medicines Agency Fimea (FIMEA/2020/002937) and the Finnish Social and Health Data Permit Authority Findata (THL/4427/14.02.00/2020).

### Tissue microarray and immunohistochemistry

Fresh slides were sectioned from the original representative tissue blocks, stained with haematoxylin and eosin (H&E), and digitized with a Pannoramic II (samples 1990–2015) or Pannoramic 250 Flash III (samples 2016–2020) (3DHISTECH, Budapest, Hungary) microscope slide scanner using a 20 × objective. Annotations for the TMAs were marked on the digitized slides with the CaseViewer 2.4 software (3DHISTECH) in accordance with the following principles: two cores from the middle of the tumour, two cores from the tumour border, two cores from the non-tumour area, and one core from the bronchus, if applicable. The TMAs were constructed with an automated TMA Grand Master tissue microarrayer (3DHISTECH) using 1 mm punches.

OTP immunohistochemical stainings were performed with a semiautomated AutoStainer instrument (Lab Vision Corp., Fremont, CA, USA). After deparaffinization, a heat-induced antigen retrieval at pH 6 was used before incubating the TMA sections with three different primary OTP antibodies: polyclonal OTP from Sigma-Aldrich (HPA059342, dilution 1:200) and two monoclonal OTP antibodies from Atlas Antibodies (CL11222 [AMAb91695] and CL11225 [AMAb91696], dilutions 1:200). EnVision FLEX + Mouse (LINKER) (Dako, Agilent Pathology Solutions, Santa Clara, CA, USA) was used for signal amplification for both monoclonal antibodies. Antibody binding was visualized with EnVision FLEX kit (Dako). All sections were counterstained with haematoxylin.

Ki-67 immunohistochemical staining was performed in the HUS Diagnostic Centre, Department of Pathology (Helsinki, Finland) with an automated BenchMark ULTRA (Ventana Medical Systems, Inc., Tucson, AZ, USA) instrument. Primary antibody clone MIB-1 (Dako) was used with CC1 standard pretreatment.

### Scoring of staining results

Immunohistochemically stained TMA slides were imaged with a Pannoramic 250 Flash III (3DHISTECH) digital slide scanner. A 20 × objective was used for brightfield imaging. Digitized slides were scored manually by two independent raters (J.N. and S.M.) blinded to patients’ clinical data, using a SlideViewer 2.6 (3DHISTECH) software and Aiforia® platform (Aiforia Technologies, Helsinki, Finland).

**Fig. 1:**
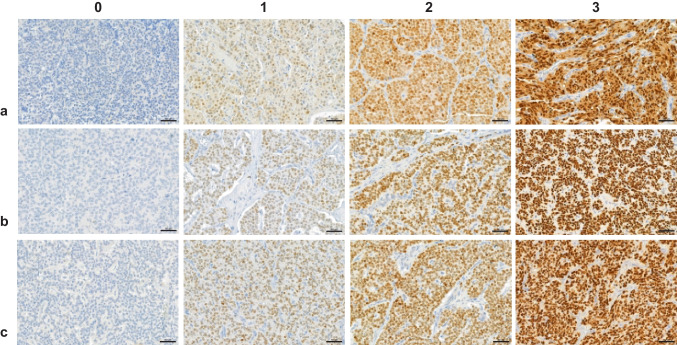
Scoring intensities for immunohistochemical OTP stainings: **a** OTP pAb, **b** CL11222 mAb, and **c** CL11225 mAb. Images were obtained with SlideViewer 2.6 (3DHISTECH, Budapest, Hungary) software with 40 × magnification (scale bar 50 µm). 0 = negative, 1 = weak, 2 = moderate, 3 = strong. *OTP*, orthopaedia homeobox protein; *pAb*, polyclonal antibody; *mAb*, monoclonal antibody

Nuclear expression of the OTP protein was evaluated by staining intensity on a scale from 0 to 3 (0 = negative, 1 = weak, 2 = moderate, and 3 = strong) (Fig. 1) and by the proportion of positive tumour cells (0%, 25%, 50%, 75%, and 100%) in each tumour core [20]. In the evaluation of heterogeneously stained tumour cores, a threshold of 40% was used to interpret the intensity. The total H-score was calculated from 2–4 different tumour cores for each sample by multiplying the staining intensity and the proportion of positive tumour cells in each core and averaging them. The staining result was classified as positive with H-score of ≥ 50, following the scoring criteria presented by Moonen et al. [20]. The results of the two raters were averaged to obtain consensus H-scores.

Ki-67 PI was analysed with deep-learning-based Aiforia software (Aiforia Technologies), as described earlier [21]. The highest Ki-67 PI of four parallel TMA spots per tumour was used in further statistical analysis.

### Statistical analysis

The association between OTP status and dichotomous clinicopathological variables was calculated using Fisher's exact test, whereas the association between continuous variables was analysed with the Mann–Whitney U test. The level of concordance between the H-scores obtained with three different OTP antibody clones was tested with intraclass correlation coefficient (ICC) two-way mixed model using the definition of absolute agreement. Agreement between raters was assessed accordingly. Cohen's kappa was calculated for dichotomous data derived from consensus H-scores.

The association of OTP expression with disease-specific survival (DSS) was evaluated using Kaplan‒Meier survival analysis with a log-rank test. DSS was determined as the time elapsed between the primary tumour surgery and death caused by PC disease. Survival probabilities were also assessed using time to progression (TTP). TTP was defined as the time elapsed from the day of the primary tumour surgery to the time of disease progression (metastasis or tumour recurrence) or death from PC tumour.

Univariable Cox survival regression analysis was applied to obtain hazard ratios (HRs) and 95% confidence intervals (CIs) for OTP status and other factors contributing to survival prognosis. Based on the univariable analysis, a multivariable Cox regression model was calculated for the most significant risk factors to evaluate the effect of other variables on the risk caused by OTP status in the patient's outcome. Testing of the Cox model assumption of a constant HR over time involved plotting the Schoenfeld residuals over time and testing for a correlation, with no relevant non-proportionality of HRs identified. The possibility of interaction terms was explored; no interactions were identified.

Two-tailed tests were used and *p* values less than 0.05 were considered statistically significant. Analyses were performed with IBM SPSS Statistics for Windows, version 29.0 (IBM Corp., Armonk, NY, USA).

## Results

### Immunohistochemical expression of OTP and association with patients' clinical data

Immunohistochemical staining with three different primary OTP antibodies (OTP pAb, CL11222, and CL11225) was performed on a total of 164 primary PC tumour samples. All OTP antibodies showed strong nuclear staining. Occasionally, low-intensity staining was observed in the cytoplasm of tumour cells. In approximately 10% of samples, a significant variation in staining intensity between the central and peripheral regions of the tumour was observed. In addition, relatively large intensity differences between the OTP clones were detected in some individual tumours (Supplementary Figure S1).

**Fig. 2:**
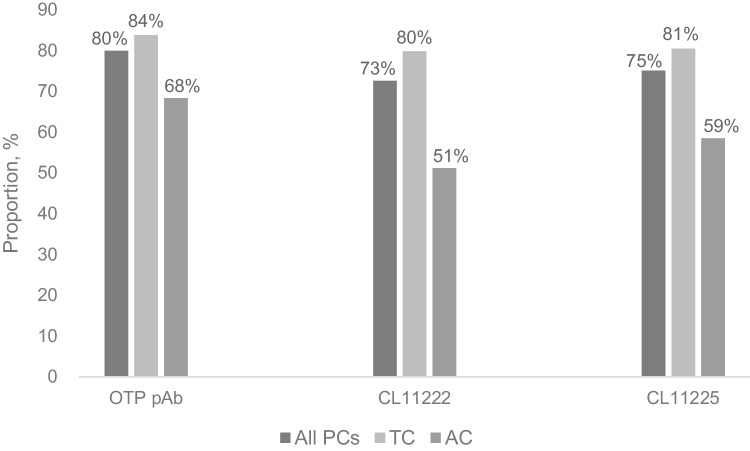
Immunohistochemical OTP expression in all PCs, TCs, and ACs for three different OTP antibody clones. H-score ≥ 50 was interpreted as positive. *OTP*, orthopaedia homeobox protein; *pAb*, polyclonal antibody; *PCs*, pulmonary carcinoids; *TCs*, typical carcinoids; *ACs*, atypical carcinoids

Through immunohistochemical analysis, TC tumours were considered significantly more frequently as positive for all OTP clones (OTP pAb, CL11222, and CL11225) than AC tumours (*p* = 0.043, *p* < 0.001, and *p* = 0.007, respectively) (Fig. 2 and Supplementary Figure S2). Furthermore, OTP positivity was associated with female sex, metastasis-free disease, and low (≤ 1%) Ki-67 PI with all antibody clones (Table 2).

**Table 2:** Association between OTP expression and clinicopathological variables

			OTP pAb			CL11222			CL11225		
*n* (%)			positive 131 (80)	negative 33 (20)	*p* value	positive 119 (73)	negative 45 (27)	*p* value	positive 123 (75)	negative 41 (25)	*p* value
Sex											
Male			36 (62)	22 (38)	< 0.001	33 (57)	25 (43)	0.002	34 (59)	24 (41)	< 0.001
Female			95 (90)	11 (10)		86 (81)	20 (19)		89 (84)	17 (16)	
Age (years)											
≤ 58			75 (86)	12 (14)	0.500	72 (83)	15 (17)	0.003	73 (84)	14 (16)	0.007
> 58			56 (73)	21 (27)		47 (61)	30 (39)		50 (65)	27 (35)	
Tumour size (cm) ^a b^											
			127	33	0.020	115	45	0.177	119	41	0.073
		Median	1.50	2.00		1.50	1.90		1.50	2.00	
		IQR	1.20	1.65		1.20	2.00		1.20	1.70	
Subtype											
TC			103 (84)	20 (16)	0.043	98 (80)	25 (20)	< 0.001	99 (81)	24 (20)	0.007
AC			28 (68)	13 (32)		21 (51)	20 (49)		24 (59)	17 (42)	
Nodal involvement at dg ^a^											
Yes			16 (80)	4 (20)	1.000	11 (55)	9 (45)	0.105	12 (60)	8 (40)	0.106
No			115 (80)	29 (20)		108 (75)	36 (25)		111 (77)	33 (23)	
Metastatic disease											
Yes			18 (60)	12 (40)	0.005	13 (43)	17 (57)	< 0.001	14 (47)	16 (53)	< 0.001
No			113 (84)	21 (16)		106 (79)	28 (21)		109 (81)	25 (19)	
Ki-67 proliferation index											
≤ 1%			105 (81)	24 (19)	0.350	99 (77)	30 (23)	0.032	101 (78)	28 (22)	0.078
> 1%			26 (74)	9 (26)		20 (57)	15 (43)		22 (63)	13 (37)	

^a^, Missing data excluded from analyses; ^b^, Mann–Whitney U test was used to obtain *p* values. *OTP*, orthopaedia homeobox protein; *pAb*, polyclonal antibody; *IQR*, inter quartile range; *TC*, typical carcinoid; *AC*, atypical carcinoid; *dg*; diagnosis

### OTP expression and patient outcome

Of the 164 PC tumour patients, 14 died as a result of PC disease. Of these, nine patients had an AC tumour and five a TC tumour. The average survival time for patients who died due to PC was 5.7 years (median 3.6 years; range 1.1–24.6 years). The average follow-up time for all patients was 14.3 years (median 13.2 years; range 1.1–32.1 years).

**Fig. 3:**
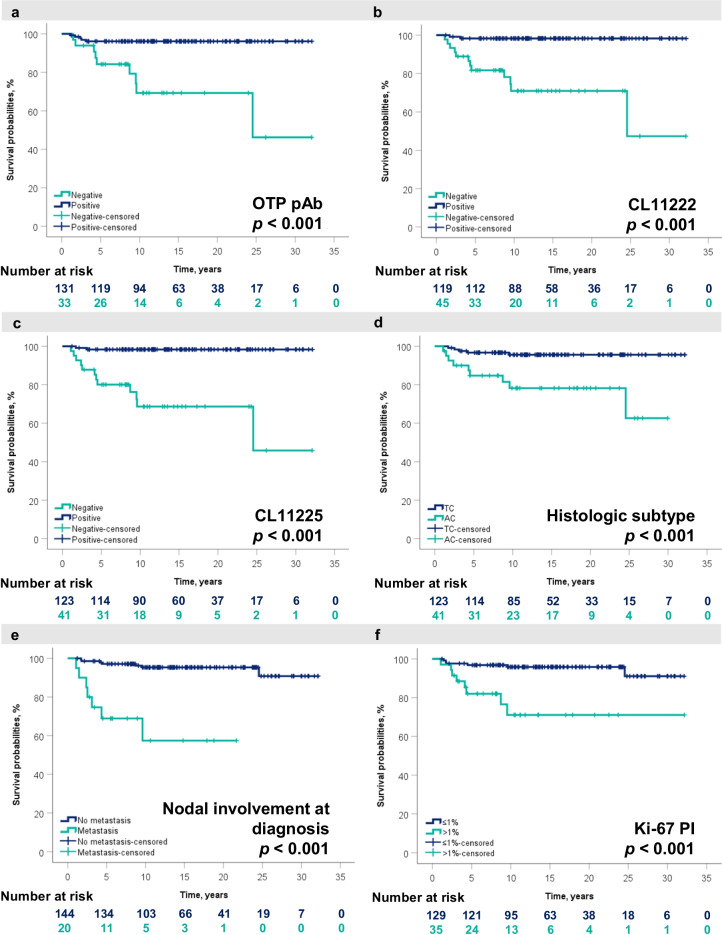
DSS probabilities for all PC tumour patients analysed with the Kaplan–Meier method based on OTP expression with three different antibody clones: **a** OTP pAb, **b** CL11222 mAb, and **c** CL11225 mAb and additionally for **d** histological subtype, **e** nodal involvement at diagnosis, and **f** Ki-67 PI. *p* values were obtained with a log-rank test. In **a-c**, blue lines indicate high protein expression, and green lines represent decreased protein expression. In **d-f**, green lines indicate AC, metastatic disease, and Ki-67 PI > 1%, while blue lines represent TC, metastatic-free disease, and Ki-67 PI ≤ 1%. *DSS*, disease-specific survival; *PC*, pulmonary carcinoid; *TC*, typical carcinoid; *AC*, atypical carcinoid; *OTP*, orthopaedia homeobox protein; *pAb*, polyclonal antibody; *mAb*, monoclonal antibody; *PI*, proliferation index

According to Kaplan–Meier survival analysis, absence of OTP expression was associated with shorter DSS: *p* values for all three OTP clones were < 0.001 (Fig. 3A-C). Patients with absence of OTP expression had a five-year DSS of 78.8% (pAb), 73.3% (CL11222), and 75.6% (CL11225). Patients with OTP expression had a five-year DSS of 90.8% (pAb), 94.1% (CL11222), and 92.7% (CL11225). The Kaplan–Meier model was also used to visualize the impact of known poor outcome factors such as AC subtype, lymph node involvement at diagnosis, and Ki-67 PI > 1% (all *p* values < 0.001) (Fig. 3D-F).

Kaplan–Meier analysis was also performed on TTP. Of all patients, 21 had metastatic or recurrent PC tumour or had encountered disease-specific death. The average time to progression or time to disease-specific death was 4.2 years (median 2.4 years; range 0.2–19.05 years). The Kaplan–Meier model showed a significant association between absence of OTP expression and disease progression; *p* values for all three OTP clones were < 0.001 (Supplementary Figure S3 A-C). Based on the analysis, AC subtype (*p* = 0.009; Supplementary Figure S3 D), lymph node involvement at diagnosis (*p* < 0.001; Supplementary Figure S3 E), and Ki-67 PI > 1% (*p* < 0.001; Supplementary Figure S3 F) were also significant contributors to disease progression.

A univariable Cox survival regression model was used to evaluate the risk caused by the absence of OTP expression in DSS and disease progression. Through univariable regression analysis, absence of OTP expression was associated with an increased risk of shorter survival and progression of PC disease (*p* values for all OTP clones < 0.001; Supplementary Table S1). Furthermore, univariable analysis showed that the AC subtype, metastatic disease, Ki-67 PI > 1%, and larger tumour size increased the risk of disease-specific death and disease progression (all *p* values < 0.05). However, other typical risk factors, such as sex and age, were not associated with the risk of worse outcome.

The risk factors that showed significance in the univariable Cox regression analysis were further tested using a multivariable Cox regression model. Based on multivariable analysis, absence of OTP expression was associated with a risk of shorter DSS and PC disease progression (*p* values for all three OTP clones < 0.001; Table 3). Lymph node involvement at the time of diagnosis also proved to be an independent risk factor (all *p* values ≤ 0.003). However, other risk factors demonstrated by the univariable model, such as AC subtype and Ki-67 PI > 1%, did not significantly increase the risk of poor outcome in the multivariable model.

**Table 3:** Analysis of potential risk factors for disease-specific death and disease progression using multivariable cox regression model. Each OTP clone was analysed separately in combination with the most significant clinicopathological variables

	DSS (No. of events: 14)			TTP (No. of events: 21)		
Variable	HR	95% CI	*p* value	HR	95% CI	*p* value
**OTP pAb**						
(Negative *vs.* Positive)	7.1	2.2–22.9	0.001	5.9	2.3–15.4	< 0.001
Histologic subtype (AC *vs.* TC)	1.8	0.5–6.6	0.380	0.9	0.3–2.5	0.770
Nodal involvement at dg ^a^ (Yes *vs.* No)	7.6	2.2–27.0	0.002	9.2	3.2–26.0	< 0.001
Ki-67 proliferation index (> 1% *vs.* ≤ 1%)	3.6	1.1–11.8	0.036	2.5	1.0–6.6	0.053
**CL11222**						
(Negative *vs.* Positive)	13.3	2.8–63.9	0.001	13.3	4.3–41.5	< 0.001
Histologic subtype (AC *vs.* TC)	1.6	0.5–5.4	0.461	0.5	0.1–1.5	0.210
Nodal involvement at dg ^a^ (Yes *vs.* No)	6.1	1.9–20.2	0.003	11.1	3.6–33.6	< 0.001
Ki-67 proliferation index (> 1% *vs.* ≤ 1%)	3.0	0.9–10.4	0.085	2.3	0.8–6.1	0.107
**CL11225**						
(Negative *vs.* Positive)	17.0	3.7–79.0	<0.001	11.5	4.0–33.0	< 0.001
Histologic subtype (AC *vs.* TC)	1.8	0.6–6.1	0.315	0.6	0.2–1.9	0.403
Nodal involvement at dg ^a^ (Yes *vs.* No)	6.6	2.0–21.7	0.002	10.2	3.4–30.5	< 0.001
Ki-67 proliferation index (> 1% *vs.* ≤ 1%)	3.7	1.1–12.6	0.039	2.6	1.0–6.8	0.058

^a^, Missing data excluded from analyses. *DSS*, disease-specific survival; *TTP*, time to progression; HR, hazard ratio; *CI*, confidence interval; *OTP*, orthopaedia homeobox protein; *pAb*, polyclonal antibody; *AC*, atypical carcinoid; *TC*, typical carcinoid; *dg*; diagnosis

### Level of agreement between raters and three different OTP antibody clones

Interrater reliability between the two raters was tested with ICC. Concordance between the H-scores of the two raters for all three OTP clones (OTP pAb, CL11222, and CL11225) was excellent. The corresponding ICC values were 0.971 (95% CI 0.958–0.980), 0.983 (95% CI 0.977–0.987), and 0.973 (95% CI 0.949–0.984).

Variability between the OTP antibody clones was assessed with ICC and Cohen's kappa analyses. Between the polyclonal (OTP pAb) and the two monoclonal (CL11222 and CL11225) OTP antibodies, results indicated a good or excellent reliability (ICC 0.831, 95% CI 0.372–0.929, *κ* = 0.800 and ICC 0.926, 95% CI 0.800–0.963, *κ* = 0.861, respectively). Between the two monoclonal antibody clones, the level of agreement was substantial to almost perfect with both tests (ICC 0.951, 95% CI 0.843–0.978, *κ* = 0.937).

## Discussion

We investigated the association of OTP expression with clinical parameters in series of 164 PC tumour patients. Furthermore, we compared the performance of three different OTP antibody clones. Based on our results, the absence of nuclear OTP expression is associated with AC subtype, metastatic disease, and adverse disease outcome. We also confirm that the results obtained with the two monoclonal OTP clones are in agreement with the results of the polyclonal antibody. Clone CL11225 behaved more like the polyclonal antibody than CL11222. A similar observation, albeit with a different reference polyclonal antibody, was made by Moonen et al. in their publication where they introduced monoclonal antibodies for the first time [20].

With all three antibodies, 80–84% of TC tumours expressed OTP, while only 51–68% of AC tumours showed OTP expression. This is in line with previous studies that report 77–89% of TCs and 43–76% of ACs to be OTP positive [10–13, 16, 22]. However, with polyclonal OTP antibody, more positivity was observed in all PCs, TCs, and ACs than with the two monoclonal antibodies. This may be because polyclonal antibody targets multiple epitopes in the human OTP sequence, reducing its reliability. On the other hand, polyclonal OTP might also be more reliable due to a more sensitive reactivity. While monoclonal antibodies may lose their specific epitope in tissue processing or storage, polyclonal antibody has targets left. This presents a general limitation in OTP immunohistochemistry since no internal positive control is available in lung samples. However, ICC scores indicate that the reliability of the staining result is better with the two monoclonal antibodies than with the polyclonal antibody used as a reference. Moreover, based on our findings, OTP expression cannot be reliably used to differentiate between TC and AC tumours. In OTP expression demonstrated with immunohistochemistry, more than 80% of the TCs and 50% of the ACs showed positivity for the protein.

The utility of immunohistochemical OTP staining in preoperative biopsy diagnosis was investigated by Naso et al. [23] and Naves et al. [24]. Naso et al. found that approximately 4% of biopsy/cytology specimens showed focal expression of OTP on immunohistochemical staining, indicating tumour heterogeneity [23]. Additionally, Naves et al. concluded that OTP expression cannot be reliably interpreted from biopsies alone and should be approached with great caution, as false negativity were observed in 3.6% of the studied biopsy-resection pairs [24]. The discrepancies in OTP staining between biopsy and resection specimens may be due to intra-tumoural variations in protein expression, as observed in this study. Based on our findings, the immunohistochemical staining patterns with OTP antibodies were mostly homogeneous for those tumour samples that were clearly positive or negative. However, there were several specimens in which the staining pattern was highly heterogeneous inside the tumour core and between the central and peripheral regions of the tumour. In these cases, a heterogeneous staining result was observed with all three antibody clones, ruling out the possibility of a staining artefact. The pre-analytical handling and variations of the tissue samples may interfere with immunohistochemical OTP staining intensities, as Naves et al. suggested [24], but variation may also be due to cell hypoxia or nuclear antigen decay caused by oxidation or storage conditions of the archival tumour specimens [25].

In the current version of the WHO classification, co-expression of nuclear OTP and CD44 is a prognostic factor for PC tumour patients [2]. Our results confirm this for the OTP since absence of OTP expression was associated with metastatic disease and shorter DSS. Moreover, in our series, absence of OTP expression was associated with an increased risk of shorter survival and progression of PC disease.

Although our results, as well as most of the literature [11–14], support the idea that the absence of OTP is an adverse prognostic indicator of PC disease, one very recent study argues otherwise. Çetin et al. [26] reported that immunohistochemical OTP expression had no association with PC subgroups, disease recurrence, or survival. This radical difference in their results compared with other studies may have a few possible explanations. First, their study included 110 PC tumours that were classified into five different groups according to OTP staining intensity (0- + 4) [26]. Because of this stratification, the number of tumours in each subgroup was relatively low, which may have caused the difference to our results. Moreover, the immunogenic region of the primary polyclonal antibody used by Çetin et al. (Invitrogen PA5-31,513) [26], targets region within N-terminal amino acids 1–159 of the human OTP sequence (total protein length 325 amino acids). The polyclonal OTP antibody used in our study (Sigma-Aldrich HPA059342) targets amino acids 227–325 at the C-terminal end. This discrepancy between results obtained with different polyclonal antibodies rightly emphasizes the importance of antibody sensitivity and specificity that monoclonal antibodies with single binding sites can provide.

The strength of our study is that we utilized FFPE primary tumour samples from a highly representative cohort of well-characterized PC tumour patients. The patients' clinical follow-up and survival data were up-to-date, and the classifications of all PC tumours were re-evaluated according to the latest WHO classification by an expert pathologist. Although the study utilized TMA technique instead of whole sections, a comprehensive number of tissue punches from different areas of the tumour were obtained. The study was performed using three different OTP antibody clones, and the level of agreement obtained with these was excellent. Similarly, the concordance of the scoring by two experienced independent raters was also close to perfect. Despite a long follow-up period, the numbers of disease-specific deaths and metastatic diseases remained low, which can be considered a limitation of this study. Automated image analysis tools were not exploited in this study for scoring OTP stainings, but their utilization would be reasonable in the future to achieve more objective and exact numerical results.

## Conclusions

The novel monoclonal OTP antibodies behave similarly to the formerly used polyclonal antibody, enabling the implementation of OTP staining in routine diagnostics. While immunohistochemical OTP status cannot be used for subtyping PC tumours reliably, the absence of OTP expression is a strong indicator for aggressive disease progression and shorter disease-specific survival in PC tumour patients. Thus, OTP immunohistochemistry should be used in PC tumour diagnostics; it identifies patients at risk for tumour relapse who would need an intensified follow-up.

## Supplementary Information

Below is the link to the electronic supplementary material.

Supplementary file1 (PDF 223 KB)

Supplementary file2 (PDF 78 KB)

Supplementary file3 (PDF 211 KB)

Supplementary file4 (DOCX 17 KB)

## Data Availability

Authors have no right to share information collected from patients or raw data, but it can be applied through Helsinki Biobank. Data: contact Helsinki Biobank, E-mail: biobank.inquiries@hus.fi.

## Declarations

The authors are accountable for all aspects of the work in ensuring that questions related to the accuracy or integrity of any part of the work are appropriately investigated and resolved. This study was conducted in accordance with the Declaration of Helsinki (as revised in 2013).
