# Supplementary material for: Absence of orthopaedia homeobox protein (OTP) expression is associated with disease spread and adverse outcome in pulmonary carcinoid tumour patients
Source: Virchows Arch. 2024 Jun 19;486(4):675–85. doi: 10.1007/s00428-024-03847-z (PMC12018497; doi:10.1007/s00428-024-03847-z)
Supplement: Supplementary file 1 — Supplementary file1 (PDF 223 KB) [file 428_2024_3847_MOESM1_ESM.pdf]

Absence of orthopaedia homeobox protein (OTP) expression is associated with disease spread and adverse outcome in pulmonary carcinoid tumour patients

Virchows Archiv

Jenni Niinimäki*, Sanna Mononen*, Tuomas Kaprio, Johanna Arola, and Tiina Vesterinen *) shared first authorship

Corresponding author:
Jenni Niinimäki, Department of Pathology, University of Helsinki and Helsinki University Hospital, Haartmaninkatu 3, FI-00014 University of Helsinki, Finland, E-mail: jenni.e.niinimaki@helsinki.fi

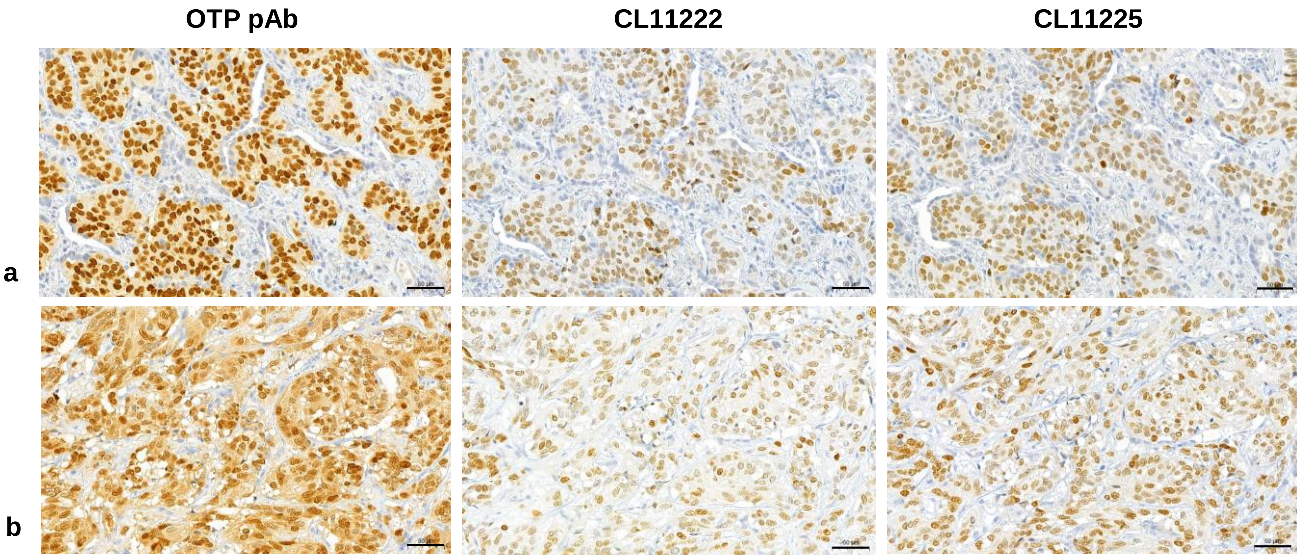

Supplementary Figure S1. Variation of immunohistochemical staining within the same tissue microarray core with three different OTP antibody clones shown in **a** TC tumour and **b** AC tumour. Images were obtained with SlideViewer 2.6 (3DHISTECH) software with 40x magnification (scale bar 50 µm). *OTP*, orthopaedia homeobox protein; *pAb*, polyclonal antibody; *TCs*, typical carcinoids; *ACs*, atypical carcinoids
