# Supplementary material for: Absence of orthopaedia homeobox protein (OTP) expression is associated with disease spread and adverse outcome in pulmonary carcinoid tumour patients
Source: Virchows Arch. 2024 Jun 19;486(4):675–85. doi: 10.1007/s00428-024-03847-z (PMC12018497; doi:10.1007/s00428-024-03847-z)
Supplement: Supplementary file 3 — Supplementary file3 (PDF 211 KB) [file 428_2024_3847_MOESM3_ESM.pdf]

Absence of orthopaedia homeobox protein (OTP) expression is associated with disease spread and adverse outcome in pulmonary carcinoid tumour patients

Virchows Archiv

Jenni Niinimäki*, Sanna Mononen*, Tuomas Kaprio, Johanna Arola, and Tiina Vesterinen *) shared first authorship

Corresponding author:
Jenni Niinimäki, Department of Pathology, University of Helsinki and Helsinki University Hospital, Haartmaninkatu 3, FI-00014 University of Helsinki, Finland, E-mail: jenni.e.niinimaki@helsinki.fi

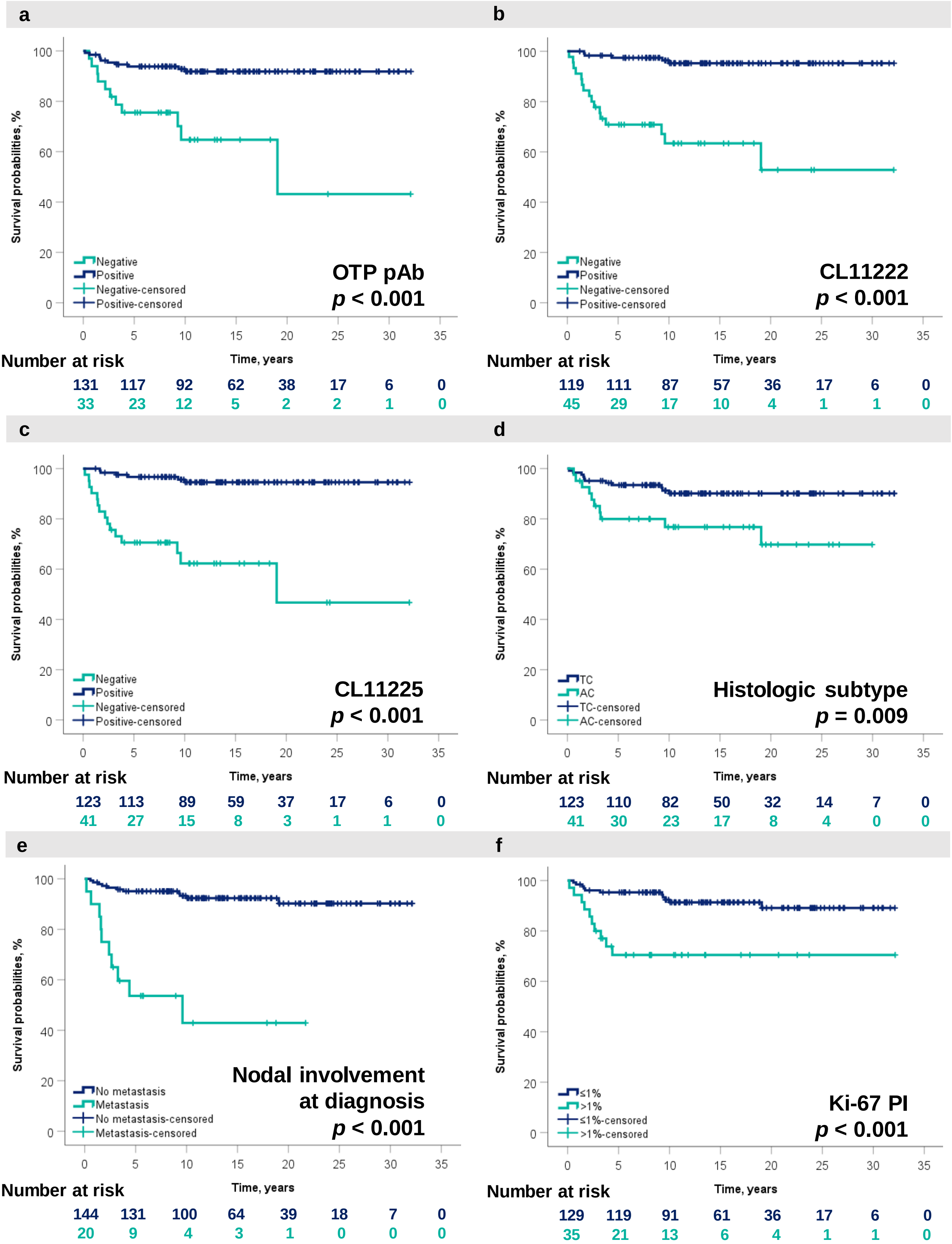

Supplementary Figure S3. Kaplan-Meier analysis performed with TTP for all PC tumour patients based on OTP expression with three different antibody clones: **a** OTP pAb, **b** CL11222 mAb, and **c** CL11225 mAb, and additionally for **d** histological subtype, **e** nodal involvement at diagnosis, and **f** Ki-67 PI. *p* values were obtained with a log-rank test. In **a-c**, blue lines indicate high protein expression, and green lines represent decreased protein expression. In **d-f**, green lines indicate AC, metastatic disease, and Ki-67 PI >1%, while blue lines represent TC, metastatic-free disease, and Ki-67 PI ≤1%. *TTP*, time to progression; *PC*, pulmonary carcinoid; *TC*, typical carcinoid; *AC*, atypical carcinoid; *OTP*, orthopaedia homeobox protein; *pAb*, polyclonal antibody; *mAb*, monoclonal antibody; *PI*, proliferation index
