# Supplementary material for: Absence of orthopaedia homeobox protein (OTP) expression is associated with disease spread and adverse outcome in pulmonary carcinoid tumour patients
Source: Virchows Arch. 2024 Jun 19;486(4):675–85. doi: 10.1007/s00428-024-03847-z (PMC12018497; doi:10.1007/s00428-024-03847-z)
Supplement: Supplementary file 4 — Supplementary file4 (DOCX 17 KB) [file 428_2024_3847_MOESM4_ESM.docx]

**Absence of orthopaedia homeobox protein** **(OTP) expression is associated with disease spread and adverse outcome in pulmonary carcinoid tumour patients**

*Virchows Archiv*

Jenni Niinimäki^*^, Sanna Mononen^*^, Tuomas Kaprio, Johanna Arola, and Tiina Vesterinen

^*^) shared first authorship

**Corresponding author:** Jenni Niinimäki, Department of Pathology, University of Helsinki and Helsinki University Hospital, Haartmaninkatu 3, FI-00014 University of Helsinki, Finland

E-mail: jenni.e.niinimaki@helsinki.fi

**Supplementary Table S1** Analysis of potential risk factors for disease-specific death and disease progression using a univariable cox survival regression model

|  | DSS  (No. of events: 14) | | | TTP  (No. of events: 21) | | |
| --- | --- | --- | --- | --- | --- | --- |
| Variable | HR | 95% CI | *p* value | HR | 95% CI | *p* value |
| OTP pAb  (Negative *vs.* Positive) | 8.3 | 2.8–24.9 | <0.001 | 5.6 | 2.4–13.3 | <0.001 |
| CL11222  (Negative *vs.* Positive) | 20.0 | 4.5–90.1 | <0.001 | 11.4 | 4.2–31.3 | <0.001 |
| CL11225  (Negative *vs.* Positive) | 22.4 | 5.0–100.6 | <0.001 | 10.4 | 4.0–27.0 | <0.001 |
| Histologic subtype  (AC *vs.* TC) | 6.0 | 2.0–17.8 | 0.001 | 3.0 | 1.3–7.0 | 0.013 |
| Nodal involvement at dg ^a^  (Yes *vs.* No) | 79.9 | 10.4–613.5 | 0.002 | 163.7 | 21.7–1234.7 | <0.001 |
| Metastatic disease  (Yes *vs.* No) | 11.9 | 3.9–35.8 | <0.001 | 10.4 | 4.3–25.1 | <0.001 |
| Ki-67 proliferation index  (>1% *vs.* ≤1%) | 6.8 | 2.3–20.0 | <0.001 | 4.3 | 1.8–10.3 | <0.001 |
| Sex  (Male *vs.* Female) | 1.9 | 0.7–5.4 | 0.235 | 1.7 | 0.7–4.1 | 0.209 |
| Age (years)  (≤58 *vs.* >58) | 0.9 | 0.3–2.7 | 0.897 | 1.2 | 0.5–3.0 | 0.651 |
| Tumour size (cm) ^a^ | 1.0 | 1.0–1.1 | 0.038 | 1.0 | 1.0–1.1 | 0.002 |
|  |  |  |  |  |  |  |

^a^, Missing data excluded from analyses; *DSS*, disease-specific survival; *TTP*, time to progression; *HR*, hazard ratio; *CI*, confidence interval; *OTP*, orthopaedia homeobox protein; *pAb*, polyclonal antibody; *AC*, atypical carcinoid; *TC*, typical carcinoid; *dg*; diagnosis
